# Expression of Stem Cell Markers in Primo Vessel of Rat

**DOI:** 10.1155/2013/438079

**Published:** 2013-07-31

**Authors:** Eun Seok Park, Jeong Hoon Lee, Won Jin Kim, Jinbeom Heo, Dong Myung Shin, Chae Hun Leem

**Affiliations:** ^1^Department of Physiology, University of Ulsan College of Medicine, 88 43-Gil Olympic-Ro, Songpa-gu, Seoul 138-736, Republic of Korea; ^2^Department of Medicine, Graduate School, University of Ulsan, 88 43-Gil Olympic-Ro, Songpa-gu, Seoul 138-736, Republic of Korea

## Abstract

Accumulating line of evidence support that adult tissues contain a rare population of pluripotent stem cells (PSCs), which differentiate into all types of cells in our body. Bonghan microcell (primo microcells (PMCs)) discovered in 1960s was reported to have a pluripotency like a stem cell *in vivo* as well as *in vitro* condition. Here, we describe the detailed morphology and molecular features of PMCs. PMCs reside in Bonghan duct (primo vessel (PV)) reported as a corresponding structure of acupuncture points and meridian system. We found that PMCs were frequently observed in the liver surface of the rat between 300 g and 400 g from April to June, suggesting that the their detection frequency depends on the weight, the season, and the organ of rat. As reported, PMCs freshly isolated from PVs were spherical ~1-2 **μ**m microsized cells. In contrast, a unique bithread or budding-shaped PMCs emerged during tissue culture around 8 days. RT-PCR analysis demonstrated that PVs-derived cells express the *Oct4*, the most important PSCs gene, in addition to several PSCs markers (*Sox2*, *Stella*, *Rex1*, and *Klf4*). Thus, we for the first time provide the evidence about Oct4-expressing stem-like characteristics for cells resident in PVs, a possible novel stem cell enriched niche.

## 1. Introduction

Continuous tissue and organ regeneration is one of the important homeostatic mechanisms of the multicellular organism. Homeostasis of adult tissues is regulated by a population of stem cells, which replace cells used up during life by undergoing self-renewal and maintaining their own pool. Stem cells are guardians of tissue/organ integrity and regulate the life span of an adult organism [1]. The most central stem cells, from a regenerative potential point of view, are pluripotent stem cells (PSCs). According to their definition, PSCs give rise to cells from all three germ layers *in vitro* and *in vivo* condition [2]. In contrast to differentiated somatic cells, PSCs commonly express pluripotent core transcription factors (TFs) such as *Oct4*, *Nanog*, and *Sox2* that are essential to maintain their pluripotent state [2].

Typically, PSCs have been established from embryonic tissue (e.g., embryonic stem cells (ESCs)) and by the ectopic expression of reprogramming factors into the terminally differentiated adult cells (e.g., inducible PSC). However, recent evidence has accumulated demonstrating that PSCs may reside in adult tissues and are able to differentiate into tissue-committed stem cells (TCSCs) [1]. These cells have been variously described in the literature as (i) multipotent adult progenitor cells (MAPCs), (ii) marrow-isolated adult multilineage-inducible (MIAMI) cells, (iii) multipotent adult stem cell (MASCs), (iv) OmniCytes, (v) Dot cell, or (vi) very small embryonic-like (VSEL) stem cell [3–8]. It has been suggested that all these cells, described by different investigators as various names, could be closely related or could be overlapping stem cell populations. Thus, exploring their relationship could advance our understanding of biological process for their pluripotency and differentiation. 

 When adult stem cell research began in the 1960s, Kim claimed to discover the structures, Bonghan duct (primo vessel (PV)), and corpuscle (primo node), and reported as a corresponding structure of acupuncture points and meridian system [9]. In succession, he reported a Sanal (primo microcell (PMC)) which was spherical shape with the size of 0.8~2.4 *μ*m containing DNA and claimed that those cells flow in primo vessel. Most interestingly, he claimed that the Sanal had a pluripotency like a stem cell by showing the pluripotency evidenced by their dividing and differentiating into several types of tissue committed cells *in vivo* as well as *in vitro* condition [10]. Since the lack of a detailed procedure for the isolation/identification of PMCs, his results have been difficult to repeat. Thus, the Bonghan theory has been largely overlooked for many years, and PMCs were regarded just as a simple cellular debris/fragments or part of apoptotic bodies. 

With advance of modern microscopy and molecular biology technologies, several researchers recently have reported the evidence for the existence of the Bonghan system inside blood or lymphatic vessels [11, 12], on the organ surface [13], and in the brain [14]. The PMCs have been also successfully isolated as DNA containing a spherical microsized (1-2 *μ*m in diameter) cell from Bonghan systems on organ surfaces using a differential centrifugation method [15]. Observation under transmission electron (TEM) microscopy has revealed that PMCs have an inner ultrastructure of a 1.5 *μ*m sized central region and many small 50–500 nm sized granules in the peripheral region, which are distinguished from apoptotic bodies and other microorganisms [16]. While most of PMCs were round-shaped, some of them had a unique protrusion and possible proliferation feature, as protruding threads under atomic force microscopy (AFM) [16]. However, their precise cellular and molecular natures have remained to be determined. 

In this paper, we isolated the primo vessel detected in rat abdomen and tracked the cellular changes using live cell imaging. And also, we investigated whether PVs express some molecular markers for PSCs such as Oct4, Sox2, Rex1, and Stella at the mRNA level. Thus, we propose that the PVs could be a potential container for the source of Oct4 expressing adult stem cells.

## 2. Materials and Methods

### 2.1. Isolation of PVs and PMC

 Sprague-Dawley rats (specific pathogen-free rat) aged 7–10 weeks were used. Procedures involving animals and their care conformed to institutional guidelines. Rats were anesthetized with intramuscular injection (Xylazine + ketamine, 1 : 4 mixture, 0.4 mL/100 g). Under the anesthesia, the abdominal wall was carefully dissected along the linea alba without any bleeding into the abdomen because the coagulation thread was easily regarded as the primo vessel [17]. Under the stereomicroscope (XTL-5, Scienscope, USA), the organ surface was thoroughly examined to find PVs with the order of liver, stomach, spleen, small intestine, large intestine, and bladder. Even though there were many studies to report PVs, until now, the confirmation criteria of PVs *in vivo* are still obscure. Therefore, firstly, we tried to find the thread-like structure on the organ surface, which was not attached and easily lifted with the forceps. If it had a node structure, it was assumed as PVs. Secondly, on the inverted microscope, the tissues were examined whether the PMCs were incorporated in the nodes. If it had the PMCs of the size of 1~2 *μ*m, we confirmed that the isolated tissues were PVs. Sometimes, we used an alcian blue (1%) to facilitate the identification of PVs *in vivo* [18]. When the cells in PVs were cultured, high glucose DMEM (GIBCO, USA) contained 10% fetal bovine serum and 1% penicillin.

### 2.2. Reverse Transcriptase (RT) and Real-Time Quantitative PCR (RQ-PCR)

 Total RNA from the PVs was isolated using the RNeasy Mini Kit (Qiagen Inc., Valencia, CA, USA) with removal of genomic DNA using the DNA-free Kit (Applied Biosystems, Foster City, CA, USA). The mRNA (10 ng) was reverse-transcribed with TaqMan Reverse Transcription Reagents (Applied Biosystems) according to the manufacturer's instructions. The resulting cDNA PCR fragments were amplified using AmpliTaq Gold at 1 cycle of 8 min at 95°C, 2 cycles of 2 min at 95°C, 1 min at 60°C, and 1 min at 72°C and subsequently by 35 cycles of 30 s at 95°C, 1 min at 60°C, 1 min at 72°C and 1 cycle of 10 min at 72°C, by using sequence-specific primers (Table 1). All primers were designed with Primer Express software (Applied Biosystems). The PCR products were separated by 1.5% agarose gel electrophoresis. 

### 2.3. Statistical Analysis

 All the data in RQ-PCR analyses were analyzed using Student's *t* test or one-way ANOVA with Bonferroni posttests. We used the GraphPad Prism 5.0 program (GraphPad Software, La Jolla, CA, USA), and statistical significance was defined as *P* < 0.05 or *P* < 0.01.

## 3. Result and Discussion

**Figure 1:**
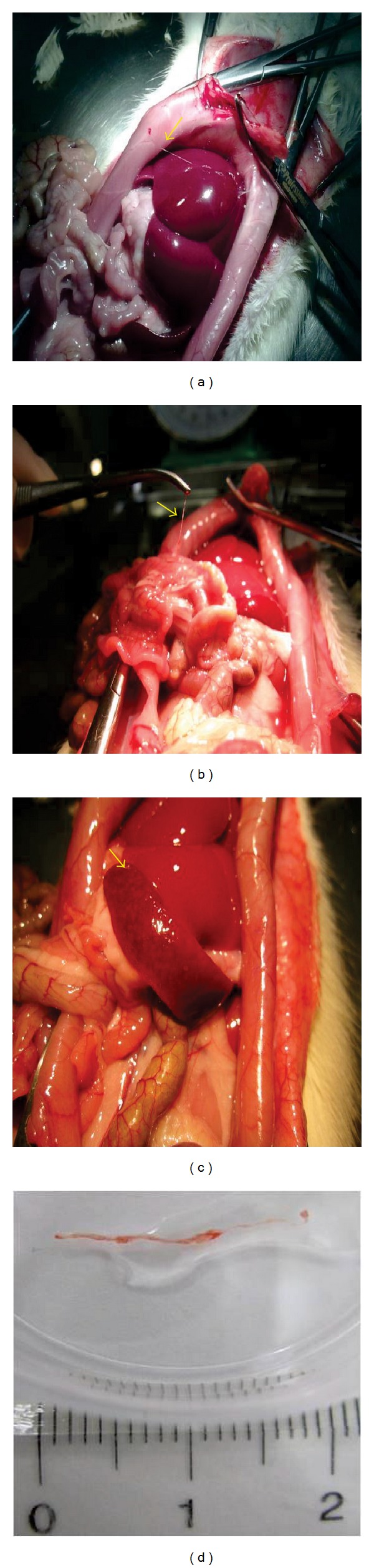
Detection of PVs on the surface of several organs. Thread-like structures presumed as PVs (arrow) were observed on organ surface such as liver (a), small intestine (b), and spleen (c). Representative photo of the isolated PV for further isolation of PMCs (d).

Figure 1 showed the thread-like structures presumed PVs on organ surface such as liver, small intestine, and spleen, respectively. As shown in Figure 1, the isolated PVs, thread-like structures were easily lifted with forceps which was the most important discrimination from blood vessel or lymphatic vessel. 

**Figure 2:**
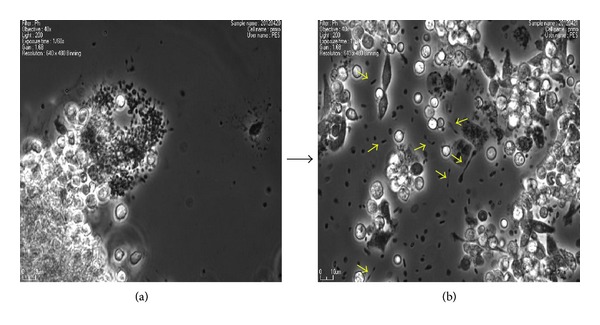
Morphologic change during tissue culture of PMCs. Representative pictures of PMCs tissue-cultured for 1 day (a) and 8 days (b). Most of PMCs at 1 day of tissue culture remained as spherical microsized cell (~1-2 *μ*m sized). Particularly, a unique bithread or budding-shaped PMCs (arrows in (b)) were observed after one week.

 Next, we examined the morphologic change during maintaining the PMCs under tissue culture condition for 8 days. Most of cultured PMCs remained as spherical microsized cell (~1-2 *μ*m sized) as similar to freshly isolated ones (Figure 2(a)). Of particular, some of the cultured PMCs showed a unique bithread shape or budding (Figure 2(b)). This transformation is similar to PMCs budding previously described by Kim [9, 10]. According to his observations, PMCs make a protrusion-like thread and produce a daughter microcell from that thread to make proliferation [9, 10]. Thus, this observation may suggest that the culture of PMCSs could promote their proliferation potency. As other expectations, this thread-like structure might be established to transfer small molecules like microvesicle [19] or to provide a polarized tension for other biological processes like migration or asymmetrical differentiation.

**Figure 3:**
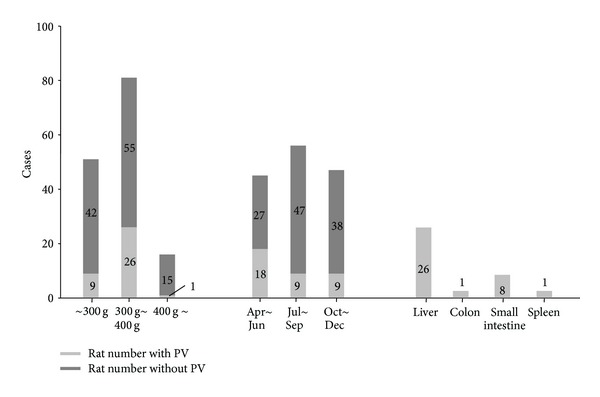
Detection rate of PMCs depends on rat weight and season. Frequency for detection of PMCs in the condition of the different weight, the season, and the organ in rat. Note that PMCs were frequently observed in the liver surface of the rat between 300 g and 400 g from April to June.

 PMCs were not identified in all rats, and the detection rate of PMCs was 24% (39 rat detected/165 rat tested). When we performed the experiments, we felt that the detection rate of PMCs was changed depending on weight and the season. We summarized the frequency for the detection of PMCs in the condition of different weight and tissue origin in rat (Figure 3). The detection rate of PMCs was the highest in the rats weighed between 300 g and 400 g, about 32%. If the weight of rat was over 400 g, the detection rate was about 6%, and if under 300 g, the detection rate was about 17%. Moreover, we found the seasonal variation of the detection of PMCs. The PMCs were more frequently found from April to June about 40%. The PMCs were most frequently found on the liver surface more than 70% and the least found on the large intestine about 5%. The earlier results suggested the guidance of the rat choice. To facilitate finding PMCs, the rats of the weight between 300 g and 400 g were used during the spring, and the surface of liver must be firstly examined. 

**Figure 4:**
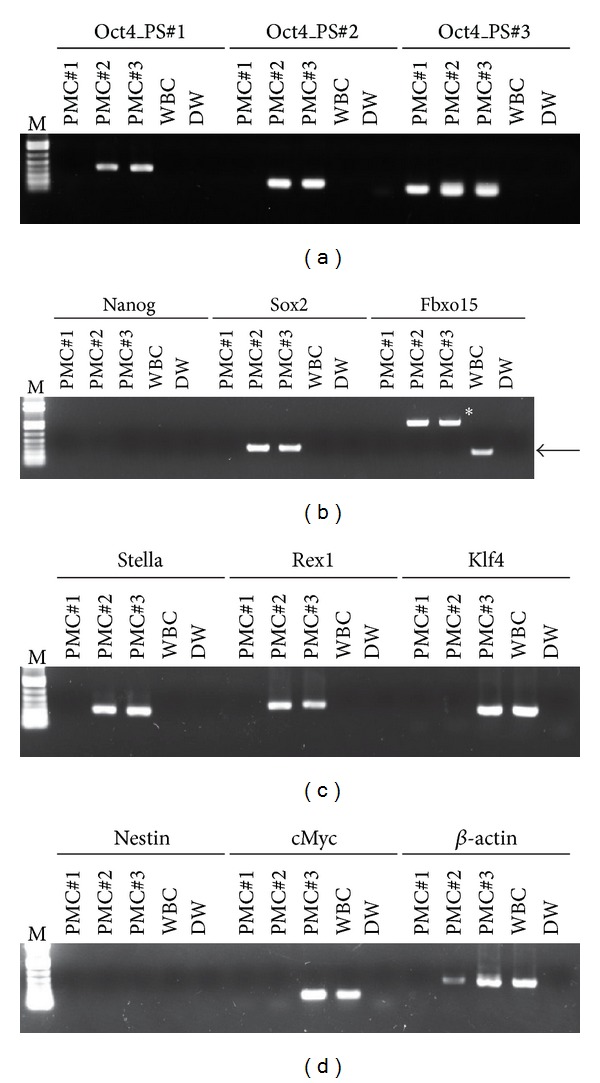
The expression of stem cell-related genes in PMCs. RT-PCR analysis of *Oct4 *(a), PSCs (b), ICM-enriched (c), neural stem cells, and *cMyc *(d) genes in freshly isolated PMCs and whole blood cells (WBC). *β*-actin was used as an endogenous housekeeping gene. Control reaction was performed without template (DW, distilled water). Arrow and asterisk in Fbxo15 part represent the expectedly sized and nonspecific PCR product, respectively. M: DNA size marker.

 To better understand the molecular insight of cells in PVs, we examined the expression of PSCs-specific genes in whole extracts from PVs. First, we tried to examine the expression of *Oct4*, the most important transcription factor for maintaining stem cell pluripotency using three independent primer sets. As shown in Figure 4(a), a PCR reaction using all the primer sets amplified transcript for *Oct4* specifically in the PV, but not in cells from adult whole blood. Since PSCs also express *Nanog*, *Sox2*, and *Fbxo15*, we evaluated their expression in PVs. They express only *Sox2*, but they do not express *Nanog* and *Fbxo15* (Figure 4(b)). The *Sox2* and *Nanog* transcripts were not expressed in whole blood cells. Next, we also examine the expression level of *Stella*, *Rex1*, and *Klf4*, highly expressed in inner cell mass primitive PSCs [20]. We noticed that PVs specifically expressed these PSC markers, which all support their pluripotent character (Figure 4(c)). In contrast, the transcript of *Nestin*, a neural stem cell marker, was not detected in any PVs tested (Figure 4(d)). Of particular, the expression of cMyc was different between PVs, representing the heterogenous features for their cell proliferation potency (Figure 4(d)). Taken together, this result provides the evidence that PVs contain the Oct4 expressing stem cell-like population, which could function as a back-up/reserve source for Oct4^+^ PSCs in adult tissues.

Lack of a detail description for the isolation and identification of PVs has been a main hurdle to get the reproducible observation of PVs. In the present study, we demonstrate that the PMCs were more frequently found from the SD rat weighed between 300 g and 400 g. Moreover, we found the seasonal variation, and most PMCs were found during the spring (April, May, and June). The liver surface was the preferred location of PMCs detection. Since we confirmed PVs when it contained microcells, PV detection rate without PMCs was not examined. Even though we did not present the detection rate during winter (from January to March), some trials during January showed that the winter season might be the worst to find PVs with PMCs (0/12 cases).

 Most of PMCs prepared from PVs show the round 1-2 *μ*m microsized morphological features, and they form the unique thread-like structure or budding during tissue culture condition. Moreover, cells resident in these PVs express the most of PSCs-specific transcripts, suggesting that they might be novel explanation about the detection of PSCs markers from adult tissues. Thus, further investigation using highly purified cells of PVs should be necessary to identify which cells in PVs could show the Oct4 expressing stem cell-like features in a molecular and cellular context, and PMCs may be the highest probable candidate having the stem cell nature.

 Present study proves that the PVs express the transcripts for PSCs (Figure 4). However, several questions remain to be addressed regarding these rare microsized cells. First, their developmental origin is unresolved. It has been considered that PSCs during embryogenesis/gastrulation may become eliminated after giving rise to TCSCs, or conversely, they may survive among TCSCs and serve as a back-up/reserve source for these cells [1]. Thus, it is important to elucidate whether PMCs are functional under steady-state conditions or are merely remnants from developmental embryogenesis that loses the potency of tissue regeneration. Second, the question is about the microsize of the PMCs. Most of PMCSs were around 1-2 *μ*m in diameter, which is smaller than normal human haploid sperm head (~2.5–3 *μ*m). This might suggest that PMCs could contain whole parts of cell organelles. Indeed, it was reported that DNA content of PMCs was around the chromosome-sized 10^8^ bps, and their DNA was fragmented [9, 10]. Thus, it is possible that PMCSs might be similar to microvesicles (MVs), small circular membrane fragments shed from the cell surface or released from the endosomal compartment. 

Due to small size, MVs also have been regarded just as simple cellular debris/fragments or part of apoptotic bodies. However, accumulating lines of evidence have reported that these tiny membrane fragments play an important and underappreciated role in cell-to-cell communication [21–23]. First, MVs may stimulate target cells directly by surface-expressed ligands acting as a kind of “signaling complex.” Second, they might transform the neighboring cells by transferring surface receptors and delivering proteins, mRNA, bioactive lipids, and even whole organelles (e.g., mitochondria). Finally, they may also serve as a vehicle to transfer infectious particles between cells such as prions or HIV. Thus, investigating the relationship between PMCs and MVs could not only advance our understanding of these microsized biocomponents but also their application in stimulating the therapeutic potency of adult stem cells. 

## 4. Conclusions

Herein, we report that PVs are frequently detected in rat grown to a weight between 300 g and 400 g. Moreover, we found the seasonal variation to detect PMC, and the spring is the best season to detect PVs. PVs were more frequently found on the liver surface than the other internal abdominal organs. And also, we provide the cellular characteristics of PMCs and the molecular characteristics of PVs. We, for the first time, provide the evidence about Oct4-expressing stem-like characteristics for cells resident in PVs, a possible novel stem cell enriched niche. Thus, functional research about their tissue regeneration potency would be essential for providing the biological significance of PVs and PMCs, especially in the field of stem cell and cancer biology.

## Figures and Tables

**Table 1:** Sequences of primers employed for RT-PCR and their anticipated PCR product size.

*β*-actin	For-CATGGCATTGTGATGGACT	427 bp
	Rev-ACGGATGTCAACGTCACACT	

cMyc	For-GGGACAGTGTTCTCTGCCTCT	199 bp
	Rev-TTCTCTTCCTCGTCGCAGAT	

Fbxo15	For-GTGGAGGAAACAGCCACA	306 bp
	Rev-ATGTGGCCAATTTTTGTCAT	

Klf4	For-CAGTCGCAAGTCCCCTCTCTC	321 bp
	Rev-CCTGTCGCACTTCTGGCACTG	

Nanog	For-GCCCTGAGAAGAAAGAAGAG	356 bp
	Rev-CGTACTGCCCCATACTGGAA	

Nestin	For-AGAGAAGCGCTGGAACAGAG	234 bp
	Rev-AGGTGTCTGCAACCGAGAGT	

Oct4_PS#1	For-GGGATGGCATACTGTGGAC	412 bp
	Rev-CTTCCTCCACCCACTTCTC	

Oct4_PS#2	For-GATGGCATACTGTGGACCT	210 bp
	Rev-TTCATATCCTGGGACTCCTCG	

Oct4_PS#3	For-GGCTGGACACCTGGCTTCAGA	204 bp
	Rev-TGGTCCGATTCCAGGCCCA	

Rex1	For-TTCTTGCCAGGTTCTGGAAGC	297 bp
	Rev-TTTCCCACACTCTGCACACAC	

Sox2	For-GGCGGCAACCAGAAGAACAG	414 bp
	Rev-GTTGCTCCAGCCGTTCATGTG	

Stella	For-TCCTACAACCAGAAACACTAG	304 bp
	Rev-GTGCAGAGACATCTGAATGG	
